# Guest Edited Collection: Epigenetics within the tumor microenvironment

**DOI:** 10.1038/s41598-022-19042-6

**Published:** 2022-09-05

**Authors:** Aamir Ahmad

**Affiliations:** Dermatology Institute and Translational Research Institute, Academic Health System, Hamad Medical Corporation, Doha, 3050 Qatar

**Keywords:** Cancer epigenetics, Molecular medicine

## Abstract

The tumor microenvironment (TME) comprises of components that exist within the immediate vicinity of tumor cells, including fibroblasts, immune cells, the extracellular matrix, and more. Significant advances have been made in recent years in our understanding of the components of TME and their mutual interactions. Part of the focus of this research has been on epigenetic events, which are increasingly being recognized for their importance in gene regulation and cancer progression. The Collection represents the gradual growth in our understanding of the overall process of how cancer progresses, along with the factors that play a decisive role in this progression. It features studies conducted on models representing many different cancers, and includes mechanistic reports conducted using appropriate in vitro models, studies that analyzed human cancer patients-derived specimens, clinical trials and, additionally, studies involving bioinformatics, metabolomics, chemical libraries screening, next-generation sequencing, and single-cell analysis approaches.

Part 1 of this Collection, ‘Recent Updates on Epigenetics in Tumor Microenvironment’, comprises papers reporting cutting-edge techniques and characterization of different factors deregulated in various human cancers, along with novel strategies for targeted delivery of antitumor therapeutics to the TME. Part 2, ‘Epigenetic events within tumor microenvironment: putative cancer therapeutic targets’, focuses on the epigenetic regulation through non-coding RNAs, methylation and acetylation, leading to dysregulated cellular growth and proliferation within the TME. Combined, the Collection provides a comprehensive snapshot of recent progress in our understanding of tumor progression from an epigenetic perspective^1–8^.

The role of immune cells, in particular, has been a hot topic of research in the context of the TME^9,10^. This is because the interactions between various cell types in the vicinity of a growing tumor create an environment that can be conducive to its continued growth. This often occurs through ‘corruption’ of immune cells which, instead of blocking tumor growth, actually start supporting it. Tumor-associated macrophages (TAMs) are a classic example of this phenomenon which, as their name suggests, associate with, and support the growth of, a tumor, instead of performing their ‘normal’ duty as macrophages to eradicate tumor cells^11^. Reports on TAMs describe their role in oral squamous cell carcinoma^12^ as well as the implications of relative abundance of M1 type macrophages favoring a TME supporting tumor progression^13^. The Collection also features an article on myeloid-derived suppressor cells (MDSCs)^14^ with focus on the levels of arginase 1 in gastric cancer patients. MDSCs are the cells that play a role in escape from immune surveillance, thus facilitating tumor progression.

Epigenetic changes mediated by non-coding RNAs are the focus of many studies in modern day cancer research. Not very long ago, non-coding RNAs were considered junk RNA^15^, but numerous investigations over last few decades have made it very apparent that non-coding RNAs play an important role in the regulation of cancer progression, drug resistance, and metastasis. Among the non-coding RNAs, micro RNAs (miRNAs) are the most widely studied subtype, followed by long non-coding RNAs (lncRNAs) and the circular RNAs (circRNAs). Accordingly, this Collection features a number of reports on miRNAs and their role in epigenetic regulation within TME. The miRNA activity explored in these reports ranges from interactions of miRNAs with transcription factors^16^, to regulation of drug response by miRNAs^17–19^, and an interesting role of miRNAs in novel anticancer activity of anesthetic propofol^20^. Even the exosome-mediated miRNA transfer is explored and reported for its effects on cancer cell invasion and therapy resistance^21^. The reports on lncRNAs MALAT1 in osteosarcoma^22^, CHRF in ovarian cancer^23^, and circRNA 101,237 in lung cancer^24^ exemplify the growing interest in epigenetic regulation through lncRNAs and circRNAs.

Methylation and acetylation are the commonly thought of as the classic epigenetic changes, and play a profound role in regulation and expression of genes. In support of this, a number of articles in the Collection touch upon these epigenetic events. One report suggests Ornithine Decarboxylase 1 as a putative master epigenetic regulator that triggers genome-wide epigenetic aberrations in urothelial cancer^25^, while another report touches upon the implications of promoter methylation of TP73 in hepatocellular and gastrointestinal cancer cell models^26^. Methylation has also been evaluated for its role in response to targeted therapies^27^. Further, not only gene methylation, but methylation of miRNA promoters also impacts cancer prognosis^28^, thus adding multiple layers to the epigenetic regulation in cancer progression. There is also a report suggesting that the simultaneous targeting of both methylation and acetylation could be an effective strategy to counter immunosuppressive TME^29^.

In addition to the identification and characterization of epigenetic events within TME for their possible diagnostic, prognostic, and therapeutic importance, it would be ideal to utilize this knowledge for novel treatment strategies. One report touches upon this topic through generation and characterization of a novel nanoparticle that is shown to exert therapeutic effects through epigenetic modulations in an osteosarcoma model^30^. In a nutshell, this Collection includes papers focused on a diverse range of topics, with each study helping us to understand the TME and the associated epigenetic regulation a little better, and progressing us towards an overarching goal of improving the diagnosis, prognosis, and treatment of cancer patients. Readers are encouraged to browse the entire Collection to appreciate the diversity and depth of topics covered.
